# Hydration Status of Elite Youth Soccer Players: Training Versus FIFA Competition

**DOI:** 10.3390/life15101546

**Published:** 2025-10-02

**Authors:** Carlos Jorquera-Aguilera, Guillermo Droppelmann-Díaz, Luis Romero-Vera, Oscar Andrades-Ramírez, César Barrientos-Bustamante, Carlos Cofré-Acevedo, Jaime Silva-Rojas, Sergio Araya-Sierralta, David Ulloa-Díaz

**Affiliations:** 1 Facultad de Ciencias, Escuela de Nutrición y Dietética, Universidad Mayor, Santiago 8580745, Chile; carlos.jorquera@mayor.cl (C.J.-A.); cesarbarrientosb@hotmail.cl (C.B.-B.); carloscofre01@gmail.com (C.C.-A.); 2 Research Center on Medicine, Exercise, Sport and Health, MEDS Clinic, Santiago 7691236, Chile; guillermo.droppelmann@meds.cl; 3 Harvard T.H. Chan School of Public Health, Boston, MA 02115, USA; 4 Facultad de Salud y Ciencias Sociales, Escuela de Ciencias de la Actividad Física, Universidad de Las Américas, Concepción 4030000, Chile; luis.romero.vera@edu.udla.cl; 5 Facultad de Educación y Ciencias Sociales, Universidad Andres Bello, Entrenador Deportivo, Concepción 4030000, Chile; o.andradesramirez@uandresbello.edu; 6 Nutrición y Dietética, Facultad de Ciencias de la Salud, Universidad de Tarapacá, Arica 1100000, Chile; jsilva@academicos.uta.cl; 7 Facultad de Humanidades y Educación, Universidad de Atacama, Copiapó 1531772, Chile; sergio.araya@uda.cl; 8 Department of Sports Sciences and Physical Conditioning, Universidad Católica de la Santísima Concepción, Concepción 4030000, Chile

**Keywords:** soccer players, hydration, urine specific gravity, water intake, national team

## Abstract

Optimal hydration is crucial for maintaining health and athletic performance in young soccer players. This requires constant monitoring by medical and sports teams during training sessions and competitions. Objective: The purpose of this study was to examine hydration status based on variations in body weight, fluid intake, and urine specific gravity during three training sessions and a FIFA competition in elite U-17 youth soccer players, national team members. Methods: Twenty-one elite soccer players, aged 17.2 ± 0.29 years, with a body weight of 72.1 ± 6.95 kg and a height of 1.80 ± 0.05 m, participated in the study. To determine hydration status, percentage weight loss, fluid intake, and urine density were measured during three training sessions and one FIFA-level competition. Results: Differences in body weight were observed in two of the training sessions, with greater variation in the competition (3.5% of BW, *p* < 0.001). Significant differences were found between weight losses in training sessions vs. matches. An increase in initial weight was associated with lower urine density. Regression coefficients showed that differences in body weight can predict urine density during training and competition (*p* < 0.05). A decrease in final body weight could be a valid indicator as a predictor of higher urinary density.

## 1. Introduction

Soccer is a team sport that involves the development of psychological, technical, tactical, and physical skills. In elite soccer players, the high demands placed on various physiological systems [1,2], metabolism [3,4], and thermoregulation [5,6,7] induce large sweat losses that alter the state of euhydration. This suggests that players need individualised hydration and rehydration strategies [8].

Fluid intake facilitates functioning both for general health and to ensure optimal athletic performance and recovery in athletes [9]. Hydration is essential for the development of multiple biological and homeostatic processes, which are often underestimated, despite being indispensable for maintaining or restoring water–electrolyte balance [10].

It has been reported that young, highly competitive athletes experience severe dehydration during sports practice [11], where physical and metabolic demands may be greater in competition than in training sessions, which has been explained by the greater hydration demands associated with growth and physical maturation in this age range [12,13]. 

Therefore, achieving a state of euhydration should be a concern in sports practice, considering that euhydration promotes digestion [14,15], the elimination of toxins, favours metabolic response, facilitates thermoregulation, joint lubrication, and neuronal conductivity [16,17,18]. Conversely, a state of hypohydration has been associated with longer recovery times [19,20], an increased risk of injury [21,22,23], and decreased athletic performance [24]. Therefore, establishing individual hydration strategies based on personal, environmental, and sporting factors would enable elite youth soccer players to achieve a minimum fluid intake.

Previous studies have analysed the effects and benefits of hydration strategies in various populations of adult soccer players. As reported in the study by Lee MY et al. (2022) [25], rehydration strategies reflect improved hydration status and predict sprint performance compared to subjective measures when playing in hot and humid environments. These results cannot be extrapolated to growing and developing adolescents [26]. It has also been reported that elite youth soccer players do not meet the recommendations for adequate fluid intake, which poses a risk of dehydration during sports practice [19,20].

Although there are guidelines on practical strategies for quantifying the risks of poor hydration and recommendations for rehydration according to the sport [21,22,23,24,25,26,27,28], it was not until the end of the previous decade that the first groups of experts were formed to propose nutrition-related strategies to promote health and improve the performance of soccer players [29]. However, it is noteworthy that current specific guidelines on fluid intake for elite youth soccer players have been developed for training and competition in controlled environments without considering the variability of training and competition conditions [30]. Furthermore, there is little research on fluid balance, sweat loss, and hydration status in elite youth soccer players during training and competition [30,31]. Urine specific gravity has been widely used as an indirect marker of hydration status, with values ≥ 1.020 g/mL being an indicator of hypohydration [32]. Studies conducted on youth soccer players have reported a high prevalence of hypohydration of 63% and up to 80% of players in training sessions [32] and competitions, and that these players are unable to replace lost sweat (~2.24 L vs. ~1.12 L intake). Santos-Sánchez et al. (2021) [33], studying a group of youth soccer players, reported an average USG of 1.021 ± 0.004 g/mL in morning urine samples during the days prior to competition, associated with sweat rates of ~582 mL/h and an average dehydration of close to 0.5% of body weight. These findings reinforce the need to monitor hydration status, considering the risk of injury and decreased performance associated with hypohydration [23,34]. Therefore, monitoring hydration status should be a concern both in training sessions and in competition, especially when players compete at an international level and represent their country [35,36].

Therefore, the purpose of the study was to examine changes in hydration status according to variations in body mass, fluid intake, and urine density in three training sessions and an official FIFA match in young players from the Chilean U-17 national team. The hypotheses were established as follows: (a) there are differences in hydration status determined by differences in body mass, fluid intake, and USG between training and a FIFA competition, and (b) differences in body mass before and after training and competition can predict hydration status by USG.

## 2. Materials and Methods

### 2.1. Participants

Twenty-one Chilean U-17 players, aged 17.2 ± 0.29 years, participated in the study. All were national team members with at least 4 years of sporting experience and were preparing for a FIFA competition. None of them had musculoskeletal injuries or competition restrictions. Anthropometric data are shown in Table 1.

### 2.2. Study Design

A repeated measures and logistic regression study was designed to meet the study objective and analyse changes in hydration status according to variations in body mass in players from the Chilean Under-17 Soccer Team. The study was approved by the Institutional Scientific Ethics Committee of the Universidad Mayor de Temuco, Chile (code 0323-2023). All procedures complied with the latest version of the Declaration of Helsinki [37]. All assessments were performed after approval by the scientific ethics committee during training sessions and before an official FIFA competition match. The research team informed the parents about the objectives of the study. All parents gave their consent for the athletes to participate, and each athlete gave their written consent. During the assessment period, no hydration or dietary recommendations were made in order to determine the athletes’ actual hydration status under normal conditions.

### 2.3. Procedure

All participants underwent four hydration status assessments. Three of these were carried out during training sessions and the fourth during a FIFA competition match. The assessments were carried out at the national training centre and at the competition stadium. The assessments during training sessions began three months before the competition and were conducted at one-month intervals. The assessment methods used to verify the players’ hydration status were percentage of body weight loss, estimated fluid intake, and urine specific gravity (USG). Table 2 shows the environmental conditions of the training sessions and the official match.

#### 2.3.1. Anthropometric Assessment

Body composition was determined according to the protocol of William D. Ross and Deborah Kerr for pentacompartmental fractionation of mass (bone, muscle, fat, residual, and skin) in AM. In all cases, ISAK standards were followed, and measurements were performed by a certified research team member (ISAK level 3). Twenty-five anthropometric measurements were taken in duplicate. Body weight was measured using a digital scale (SECA^®^ model EF211BW, Hamburg, Germany) with an accuracy of 100 g. Height was measured using a portable, collapsible SECA^®^ model 206 stadiometer with an accuracy of 0.1 cm. Eight circumference measurements (head, relaxed arm, flexed arm at tension, maximum forearm, mid-sternal chest, minimum waist, maximum hip, maximum thigh, medial thigh, and maximum calf) were recorded using a Lufkin WP-606 steel tape measure (Lufkin, TX, USA). The four large bone diameters (biacromial, transverse thoracic, anteroposterior thoracic, and biiliocrestal) were measured using a Rosscraft ^®^ Campbell 20 sliding anthropometer (Rosscraft, Surrey, BC, Canada). The two small bone diameters (humeral and femoral) were measured using a Rosscraft^®^ Campbell 10 sliding anthropometer (Rosscraft, Surrey, BC, Canada). The six skinfolds (triceps, subscapular, suprascapular, abdominal, medial thigh, and maximum calf) were measured using a Slim Guide Skinfold caliper (Creative Health Products, Plymouth, MI, USA). The sum of 4 skinfolds (biceps, triceps, subscapular, and suprascapular) and 6 skinfolds (triceps, subscapular, supraspinal, abdominal, front thigh, and calf) were considered.

#### 2.3.2. Hydration Status

To determine hydration status, percentage body weight loss (%BW), estimated fluid intake (EFI), and urine specific gravity (USG) were used. %BW was calculated using the difference in body weight before and after each training session and competition. Measurements were always taken by the same evaluator and were performed without footwear and wearing only sports trousers. In all cases, the same scale used in the anthropometric evaluation was used.

To determine hydration status before and after training and competition, players were required to provide a urine sample in an individual container of at least 0.3 mL during the morning session. USG was determined using a Robinair^®^ portable refractometer, (Robinair, Spx, Owosso, MI, USA). The samples were collected and organised by one of the researchers for subsequent analysis. In all cases, the recommendations of Casa et al. (2000) [38] and Martinho et al. (2014) [39] were followed. The following criteria were used to establish USG. A value below 1.010 g/mL indicated euhydration. A value between 1010 and 1019 mg/L was considered minimally dehydrated, a value equal to or greater than 1020 mg/L was considered dehydrated, and when it was equal to or greater than 1030 mg/L, it was considered severely dehydrated.

**Figure 1:**
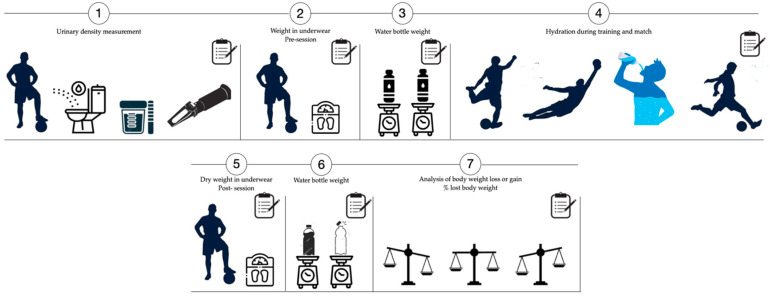
Methodological process of the study.

To control LDL during training and competition, two one-litre bottles of water were provided, which were refilled when empty for consumption ad libitum. A member of the research team recorded total fluid intake. Each bottle was labelled with a number for each player. The amount of fluid ingested was calculated by recording the difference in weight between the two bottles. An additional water dispenser was used so that participants could cool themselves by splashing their hair or skin, thus avoiding drinking the water provided. The procedure is shown in Figure 1.

### 2.4. Statistical Analysis 

All data were reduced to means, medians, and standard deviations. The Shapiro–Wilk test was performed to determine data distribution. Athlete means were recorded according to hydration level and USG category. The Friedman test for related samples and the Wilcoxon test were used to analyse differences between medians for each training session and on the day of competition for continuous variables. The Friedman test was applied to estimate differences between the ranks or positions of the data for initial body weights during training days and on the day of competition. In all studies, USG was considered an ordinal variable. Ordinal logistic regression was used, calculating the beta coefficient (β) and the standard error (SE) to evaluate the dependence of urine specific gravity on initial and final weights and fluid intake. The analysis considered USG as the dependent variable and body weight before, body weight after, and amount of fluid consumed as independent variables. A statistical significance level of *p* < 0.05 was considered using a two-tailed hypothesis. Data were recorded in Microsoft Excel and analysed using the statistical program R 4.5.1 (Great Square Root).

## 3. Results

The average values of the analysis variables are shown in Table 3.

**Figure 2:**
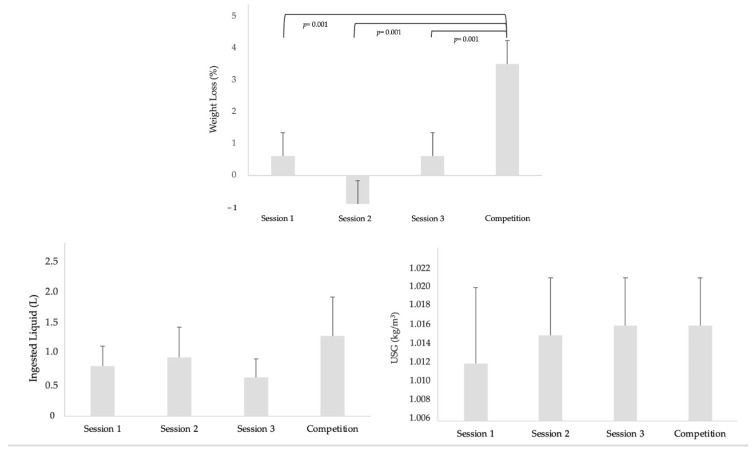
Descriptive data on ingested liquid, urine specific gravity (USG), and percentage weight loss during training sessions versus competition.

**Figure 3:**
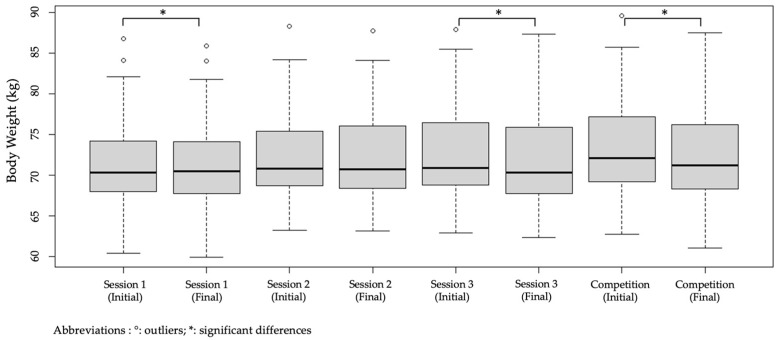
Body weight variation in young elite soccer players at the initial and final training sessions on competition day.

Significant differences (*p* ≤ 0.001) were observed in the analysis of samples related to fluid intake and weight loss. Regarding the USG variable, no significant differences were found (*p* = 0.083). In addition, significant decreases (*p* ≤ 0.001) in body mass were observed between the start and end of sessions one and three, and the match, as shown in Figure 2. When comparing the difference in body mass between sessions, changes in body mass before (*p* = 0.001) and after (*p* = 0.001) were compared. Figure 3 shows the details of the weight variations according to the training day and the official match day.

The ordinal logistic regression model (Table 4) revealed that initial body mass and final body mass are associated with USG composition, both in the first training session and in the official match. The estimated regression coefficients show that these variables predict USG behaviour. An increase in initial body mass is associated with a decrease in the probability of moving up to a higher USG category, while an increase in final weight is associated with an increase in the probability of moving up to a higher category.

## 4. Discussion

The purpose of the study was to examine changes in hydration status based on variations in body mass, fluid intake, and urine specific density during three training sessions and an official FIFA match in young soccer players from the Chilean U-17 national team. The hypothesis was that differences in body mass before and after training and competition could predict hydration status.

Our results suggest that hydration status measured by percentage weight loss, fluid intake, and USG differed between training sessions and when compared to competition. The percentage weight loss in session 1 was 0.61%, in session 3 it was 0.89%, and in SP it was 3.47%. In contrast, in session 2, players gained a small amount of weight (−0.13%), which would indicate that fluid intake exceeded losses through perspiration. 

On average, total fluid intake ranged from 0.62 L to 0.94 L in training sessions and reached up to 1.29 L in competition. In both training sessions and competition, most participants experienced some degree of minimal dehydration (USG 1.010 to 10.20) at some point. Between 38.09% and 76.19% experienced some degree of significant dehydration (SGS between 1.021 and 1.030), and between 9.52% and 1.04% experienced dehydration, with no cases of severe dehydration reported. Acute fluid losses exceeding 2–3% of body mass not only affect performance but also increase the risk of thermoregulatory overload [40], heat-related illnesses [41], renal stress [42], and cognitive impairment in young people [43]. These issues are particularly relevant for under-17 players, who may have lower tolerance and lower hydration levels compared to adults. Given that young athletes may have lower sweating efficiency, lower acclimatisation capacity, and less knowledge of hydration strategies compared to adults, these risks are increased [44].

Regression analysis determined that pre- and post-competition body weights are determinants of USG in a competition.

Dehydration has been linked to weight loss before and after training sessions [44] and competitions [45]. Da Silva et al. (2012) [31] revealed that during competitions, young players do not compensate for sweat loss by drinking fluids. These authors reported that the percentage of weight loss was close to 2%, the threshold at which a decrease in physical performance has been documented [31]. The study by Santos-Sánchez et al. (2021) [33], which evaluated the hydration status of 26 elite young soccer players (15 ± 1.2 years) during the three days prior to a competition, showed an average body weight loss of 0.5%. In our study, weight loss during training sessions was similar to that previously reported. However, during competition, body weight loss reached 3.47%, which could be explained by environmental differences between training sessions and competition or because players did not manage to recover fluid loss when the hydration strategy was the same as in training [46].

Previous studies indicate that replacing at least 70% of the fluid lost during training promotes recovery [47] by reducing the risk of fatigue [48]. Adequate hydration can prevent weight loss due to dehydration and preserve athletic performance [45,46,47,48]. Fluid intake strategies should also consider the degree of hydration prior to training sessions, competition [49], and environmental conditions [50].

In our study, despite the fact that initial hydration status during competition was better than in training sessions, the hydration strategy failed to compensate for the loss of body fluids. In a previous study, Chapelle L. et al. (2020) [32] reported a high prevalence of pre-competition hypohydration among young soccer players, ranging from 63% to 80% (USG > 1.020), both in training sessions and prior to competition. This coincides with the findings of Santos-Sánchez et al. (2021) [33] when analysing morning USG samples (1.021 ± 0.004 g/mL) from youth soccer players in the days leading up to a competition. Our findings confirm that young soccer players usually start training and competition in a state of hypohydration, and that hydration strategies do not always compensate for fluid losses, especially in competitive contexts and demanding environmental conditions. 

Studies conducted on young soccer players have shown that, prior to competitions, both in warm (≈31 °C) and temperate environments, young players tend to be hypohydrated (USG > 1.020%) [31,32]. The environmental conditions in our study varied greatly between training sessions (3–8 °C) and competition (22 °C), which could explain the greater weight loss during competition. Our results are consistent with those published by Da Silva et al. (2012) [31], who reported that, in hot competition conditions, young elite players only manage to replace an average of 50% of the fluid lost during competition, which could lead to a loss of up to 3% of body mass.

There is a high prevalence of hypohydration at the beginning of training and hypohydration states (USG ≥ 1.020) that persist after the training session [51]. All this information reinforces the need to implement individualized interventions based on biomarkers (USG, body weight variation, and/or thirst sensation), following the recent recommendations that promote the creation of personalized hydration plans for each athlete [52]. In this sense, the systematic assessment of USG in morning samples accompanied by an intake strategy based on body weight variations could significantly improve the pre-, intra-, and post-exercise hydration status, reducing fatigue and optimizing performance in young soccer players.

## 5. Conclusions

Our results demonstrate that a high percentage of elite youth soccer players, both in training and in a FIFA match, present high levels of dehydration and significant differences in percentage of weight loss, fluid intake, and USG between the two settings. Body mass changes before and after competition are determinants and have predictive value for USG in players. Specialized teams should implement practical strategies to quantify the risks of poor hydration and provide guidance on rehydration to young elite soccer players.

### 5.1. Limitations

Due to the nature of the population and the type of sample studied, these results cannot be extrapolated to other groups and should be considered with caution when implementing hydration strategies in other types of untrained populations.

### 5.2. Clinical Applications

Dehydration prior to training and competition should be a concern for medical teams, coaches, and athletes themselves. Our results can guide the development of hydration strategies. An individualised and differentiated strategy is recommended for training sessions and competitions. Monitoring body mass before and after exercise can be a valid strategy for monitoring individual hydration status in young players. These aspects are particularly relevant for under-17 players, who may have lower tolerance and lower hydration levels compared to adults. We believe that these modifications reinforce the clinical relevance of our findings.

### 5.3. Recomendaciones Para Future Research

Future research should analyse hydration levels in different types of training sessions, depending on the competition period and the training periodisation method and model structure, considering that soccer players are subjected to different stimuli depending on the above factors.

## Figures and Tables

**Table 1:** Anthropometric characteristics of the study population.

	Mean ± DS	Maximos–Minimal
BM (kg)	72.1 ± 6.95	84.00–62.30
Size (cm)	1.80 ± 0.05	1.84–1.68
BMI	23.5 ± 1.88	28.3–21.1
FM (kg)	21.7 ± 2.77	22.6–10.9
% FM	15.5 ± 2.89	26.0–17.0
MM (kg)	48.8 ± 2.51	41.1–27.1
% MM	34.4 ± 3.78	54.0–43.0
∑ 4 folds (mm)	30.8 ± 10.52	64.5–15.5
∑ 6 folds (mm)	49.0 ± 13.97	92.5–31.5
MBI	4.10 ± 0.41	5.0–3.5

Abbreviations: BM: body mass; BMI: body mass index; FM: fat mass; % FM: percentage fat mass; MM: muscle mass; % MM: percentage muscle mass; ∑: summation; MBI: muscle bone index.

**Table 2:** Environmental conditions during sessions and on the day of the competition.

Day	T°	RH	Wind Speed
Session 1	3 °C	9.00%	5.6 km/h
Session 2	8 °C	81.30%	7.4 km/h
Session 3	3 °C	93.10%	3.7 km/h
Competition	22 °C	38.00%	24 km/h

Abbreviations: T°: temperature, RH: relative humidity, °C: Celsius degrees.

**Table 3:** Average values of the analysis variables for the three training and competition sessions.

Day	Weight Loss (%)						Ingested Liquid (L)						USG (kg/m^3^)					
	Mean	±	SD	IC95%			Mean	±	SD	IC95%			Mean	±	SD	IC95%		
Session 1	0.61	±	0.60	0.32	–	0.87	0.80	±	0.32	0.66	–	0.96	1.012	±	0.008	1.00	–	1.01
Session 2	−0.13	±	0.70	−0.44	–	0.19	0.94	±	0.49	0.71	–	1.16	1.015	±	0.006	1.01	–	1.01
Session 3	0.89	±	0.76	0.53	–	1.22	0.62	±	0.30	0.47	–	0.75	1.016	±	0.005	1.01	–	1.01
Competition	3.47	±	1.05	3.00	–	3.95	1.29	±	0.62	1.00	–	1.56	1.016	±	0.005	1.01		1.01

Abbreviations: %: percentage; L: Liters; USG: urine specific gravity; kg: kilograms; m^3^: cubic meters; SD: standard deviation; IC95%: 95 percent confidence interval.

**Table 4:** Urine density according to training day and official game.

Variable	Condition	β	SE	*p*
Initial Body Mass (kg)	Session 1	−3.960	1.590	0.012 ^†^
	Session 2	0.749	1.318	0.570
	Session 3	0.395	1.010	0.695
	Competition	−4.199	1.931	0.029 ^†^
Final Body Mass (kg)	Session 1	4.009	1.601	0.012 ^†^
	Session 2	−0.656	1.325	0.621
	Session 3	−0.478	1.024	0.641
	Competition	4.142	1.945	0.033 ^†^
Liquid intake (L)	Session 1	−1.780	1.874	0.342
	Session 2	−0.092	1.441	0.949
	Session 3	0.589	1.897	0.756
	Competition	1.981	1.193	0.097 ^†^

Abbreviations: β: beta coefficient, SE: Standard error, *p*: *p*-value, †: statistical significance.

## Data Availability

The raw data supporting the conclusions of this article will be made available by the authors upon request.
